# Sex and Gender Equity in Prehospital Electrocardiogram Acquisition

**DOI:** 10.1017/S1049023X2200036X

**Published:** 2022-04

**Authors:** Neil McDonald, Nicola Little, Rob Grierson, Erin Weldon

**Affiliations:** 1. Winnipeg Fire Paramedic Service, Winnipeg, Manitoba, Canada; 2. Applied Health Sciences - University of Manitoba, Winnipeg, Manitoba, Canada; 3. Department of Emergency Medicine - University of Manitoba, Winnipeg, Manitoba, Canada; 4. Shared Health Manitoba - Emergency Response Services, Winnipeg, Manitoba, Canada

**Keywords:** 12-lead ECG, acute coronary syndrome, Emergency Medical Services, gender equity, sex

## Abstract

**Introduction:**

Research in cardiac care has identified significant gender-based differences across many outcomes. Women with heart disease are less likely both to be diagnosed and to receive standard care. Gender-based disparities in the prehospital setting are under-researched, but they were found to exist within rates of 12-lead electrocardiogram (ECG) acquisition within one urban Emergency Medical Services (EMS) agency.

**Study Objective:**

This study evaluates the quality improvement (QI) initiative that was implemented in that agency to raise overall rates of 12-lead ECG acquisition and reduce the gap in acquisition rates between men and women.

**Methods:**

This QI project included two interventions: revised indications for 12-lead acquisition, and training that highlighted sex- and gender-based differences relevant to patient care. To evaluate this project, a retrospective database review identified all patient contacts that potentially involved cardiac assessment over 18 months. The primary outcome was the rate of 12-lead acquisition among patients with qualifying complaints. This was assessed by mean rates of acquisition in before and after periods, as well as segmented regression in an interrupted time series. Secondary outcomes included differences in rates of 12-lead acquisition, both overall and in individual complaint categories, each compared between men/women and before/after the interventions.

**Results:**

Among patients with qualifying complaints, the mean rate of 12-lead acquisition in the lead-in period was 22.5% (95% CI, 21.8% - 23.2%) with no discernible trend. The protocol change and training were each associated with a significant absolute level increase in the acquisition rate: 2.09% (95% CI, 0.21% - 4.0%; P = .03) and 3.2% (95% CI, 1.18% - 5.22%; P = .003), respectively. When compared by gender and time period, women received fewer 12-leads than men overall, and more 12-leads were acquired after the interventions than before. There were also significant interactions between gender and period, both overall (2.8%; 95% CI, 1.9% - 3.6%; P < .0001) and in all complaint categories except falls and heart problems.

**Conclusion:**

This QI project resulted in an increase in 12-leads acquired. Pre-existing gaps in rates of acquisition between men and women were reduced but did not disappear. On-going research is examining the reasons behind these differences from the perspective of prehospital providers.

## Introduction

Recent research in cardiac care has identified significant differences between men and women across many outcomes. In general, women fare worse than men in the identification, treatment, and rehabilitation of both acute and chronic cardiac disease.^1–9^ Hypothesized explanations for these disparities include both sex- and gender-based differences, where sex refers to a person’s biological make-up and gender encompasses an individual’s self-identity and expression.^9–11^ While a significant body of literature exists documenting this phenomenon in both primary care and in-hospital settings, comparatively little research has examined these disparities in the prehospital environment.

Early identification of acute coronary syndrome (ACS) relies on the 12-lead electrocardiogram (ECG). As with other interventions, the indications for a 12-lead have historically relied on symptoms, especially chest pain, that were thought to be typical for men.^12,13^ Other research has emphasized the broad range of possible complaints beyond chest pain, such as dyspnea, altered mental status, upper extremity pain, syncope, dizziness, weakness, abdominal pain, nausea, vomiting, falls or collapse, and confusion or delirium.^14–16^ Whereas these signs have been characterized as “atypical” presentations more common among women, more recent research and commentary has questioned sex differences in presenting symptoms in ACS and suggested that “typical” and “atypical” are themselves misnomers: signs and symptoms suggestive of ACS should be considered without reference to preconceived notions of associated sex or gender.^17–19^

Among prehospital interventions related to ACS, the 12-lead has become a standard of care in Emergency Medical Services (EMS). In many services, early identification of an ST-elevation myocardial infarction/STEMI on a 12-lead allows crews to minimize time to definitive treatment.^20–22^ Given the potential for improved outcomes from early recognition, treatment, and transport of ACS patients by prehospital teams, it is important to examine whether those benefits are both optimized for all patients and distributed equitably between men and women. After preliminary investigation in one EMS agency, baseline rates of ECG acquisition showed room for improvement when compared to broad indications for assessment, and women were found to receive a 12-lead ECG less frequently than men across all relevant complaint categories.

These findings provided the rationale for a local two-stage quality improvement (QI) project. This project aimed to increase the rate of ECG acquisition overall and reduce the gap between men and women through two interventions. First, the formal indications for 12-lead acquisition were broadened to include non-traditional ACS symptoms. Second, this clinical and system change was followed by scenario-based training that highlighted potential sex- and gender-based differences relevant to patient care. The objective of this study is to evaluate the impact of these interventions on rates of 12-lead acquisition in the prehospital setting.

## Methods

### Context

This is a retrospective database review of all prehospital patient contacts that potentially involved assessment for ACS before and after QI interventions, from January 2018 through June 2019. The study took place in Winnipeg, Manitoba, a city of approximately 750,000 located near the east-west center of Canada. The local service provides 9-1-1 coverage within the city limits and responds to over 80,000 primary medical calls a year. It uses a tiered-response model with Basic Life Support (BLS) first responders and mixed Basic and Advanced Life Support transport capability. This study adheres to the Standards for Quality Improvement Reporting Excellence (SQUIRE 2.0) guidelines.^23^ Approval for the study was granted by the affiliated Health Research Ethics Board (HS22981 [H2019:273]).

### Terminology

Following other research in this area, this study refers to a person’s biological make-up as “sex” and their individual self-identity and expression as “gender.”^10,11^ Both concepts have been described as relevant to a patient’s risk for ACS and presentation to health care providers with signs and symptoms that warrant investigation.^9–11^ Accordingly, in-service training on presenting signs and symptoms included information on both sex and gender. However, the documentation platform in use during the study period (RescueNet; ZOLL Medical; Chelmsford, Massachusetts USA) recorded “gender” but offered only a choice between male and female for a required response, a common limitation of data systems.^24^ In practice, front-line providers take in a variety of information and cues to complete this field, including how patients refer to and present themselves, how bystanders and family refer to the patient, observed patient characteristics, and how patient information is recorded in the provincial health care database. Acknowledging a lack of non-binary options as well as limited training in this area,^24,25^ and in common with similar studies,^26^ this paper will use the term gender (woman, man) to describe how patients are documented in the case of potential ACS calls. It will follow terms in print when referring to other studies.

### Interventions

Initial tests of change and iterative discovery of the norms surrounding 12-lead acquisition identified knowledge gaps among frontline staff and specific areas for improvement. The resulting QI interventions aimed to remove inconsistencies in crews’ understanding of both the indications for and timing of 12-lead acquisition, and to encourage BLS first responders to proceed with assessment when indicated. The first intervention added revised indications for 12-lead acquisition to the ACS protocol in July 2018 (Table 1). These revisions were written by the Service Quality Branch under the supervision of the service’s medical directors and drawn from published research on the topic. Up until that time, the ACS protocol listed chest pain as the sole indication for a 12-lead ECG; in practice, all providers could interpret indications more or less broadly depending on their qualification level, time since training, experience, and personal knowledge. The second intervention occurred in the fall of 2018 and consisted of in-person, scenario-based training provided to BLS first responders to emphasize sex- and gender-based factors relevant to patient care. Lasting one or two hours, these small-group sessions were led by a clinical leader and were based on training scenarios drawn from prior cases identified by the Service Quality team. This training period lasted eight weeks. The time periods before the protocol change and after the end of training were each approximately six months.

**Table 1.** Revised Protocol Indications for 12-Lead Acquisition and Study Inclusion Criteria

	Revised Protocol	Study Inclusion
Age Category	12-Lead Indicated with Presenting Complaints	MPDS Category, Number
>30	Chest Pain	Chest Pain, 10
		Heart Problems, 19
	Short of Breath	Short of Breath, 6
	Syncope	Syncope, 31
>50	Altered Mental Status	General Illness, 26
	Weakness	Falls, 17
	Upper Extremity Pain	
>70	Abdominal Pain	Abdominal Pain, 1
All	Any Significant Risk Factors or Poor Impression	

### Measures

In the absence of any established method for retrospectively identifying prehospital patients who require a 12-lead, this study used the broad complaints listed in the revised protocol as the basis for inclusion (Table 1). Patient complaints were identified in the database of electronic patient care reports (ePCRs) by matching them with the closest corresponding dispatch code under the Medical Priority Dispatch System (MPDS; Salt Lake City, Utah USA); these codes are also listed in Table 1. Cases matching age and dispatch-code criteria were included; those that did not were excluded. Due to the broad categorization of complaint, this method would be expected to over-count the number of eligible patients (the denominator), and therefore under-estimate rates of acquisition among those at risk for ACS. It would, however, capture the broadest sample of eligible patients and provide reliable estimates of change over time – both of which are consistent with the study objective. In the local service, all medical 9-1-1 calls are assigned an MPDS code (with the exception of those transferred directly from police). The ePCR records of eligible patients were reviewed for documentation of a 12-lead ECG. Frontline crews record 12-lead acquisition either by clicking on an intervention button in the documentation software or by uploading data from the cardiac monitor. There is no way to retrospectively assess missingness in 12-lead documentation, but practice norms discussed by crews during the initial stages of the project suggest it is exceedingly rare to complete a 12-lead and not document it; if present, missingness would bias results towards lower rates of acquisition.

### Outcomes and Analysis

The primary outcome was the rate of 12-lead acquisition among patients with qualifying complaints. Secondary outcomes included differences in rates of 12-lead acquisition both overall and in individual MPDS complaint categories, each compared between men/women and before/after the interventions.

In the case of the primary outcome, rates of 12-lead acquisition were calculated for the before and after periods (mean, 95% CI) and also analyzed by segmented regression in an interrupted time series. The use of an interrupted time series analysis for the primary outcome has several benefits over a simple before-and-after comparison. Considered the most robust of quasi-experimental approaches,^27^ an interrupted time series can account for underlying trends in data, estimate the effect of each intervention, and gauge the staying-power of effects over time – all benefits particularly relevant to this project. Data were plotted on a weekly basis over 78 weeks, with a mean of 778 observations per week. Segments were defined by the implementation of the new protocol (between weeks 26 and 27) and the in-service training that occurred during weeks 48 - 55. Given gradual accumulation of trained providers and small number of observations over the training period, data within this time were censored, and level and trend changes were calculated between the beginning and end of the training.^28^ After an initial model was fit, autocorrelation was assessed using plots of the autocorrelation and partial autocorrelation functions as well as the Durbin-Watson test of residuals. The model was then re-specified with candidate autoregressive (AR) or moving average (MA) terms. Initial testing identified three models for comparison: no AR or MA terms, AR(*p*) = 5, and AR(*p*) = 7. The model with no terms was shown to have the best fit (resulting Durbin-Watson test of residuals: 1.77; P = .057). No effect of seasonality was anticipated or modelled.

Secondary outcomes were analyzed in terms of mean rates of 12-lead acquisition before and after training. In each analysis, all eligible cases were included in a linear model with time period and gender as predictor variables (main effects and interaction). Results were considered statistically significant at alpha = 0.05. All analysis was performed in R, version 4.0.4 (Foundation for Statistical Computing; Vienna, Austria).

## Results

### Primary Outcome: 12-Lead Acquisition among Patients with Qualifying Complaints

In the lead-in period prior to the protocol change, the mean rate of 12-lead acquisition among patients with qualifying complaints was 22.5% (95% CI, 21.8% - 23.2%). After training until the end of the study period, the mean rate was 27.2% (95% CI, 26.5% - 27.9%).

**Figure 1.**
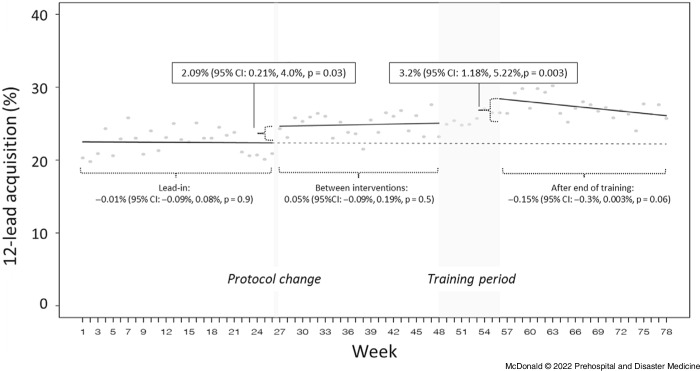
Interrupted Time Series of 12-Lead Acquisition among Patients with Qualifying Complaints. Note: Weekly data from January 2018 through June 2019 (78 weeks), with interventions and study periods as marked. Values above the chart show absolute level changes associated with each intervention. Values below correspond to trend changes during each study period relative to the period before.

The interrupted time series for 12-lead acquisition among this group is summarized in Figure 1. Of note, each intervention was associated with a significant absolute level increase in the acquisition rate. In the case of the revised protocol, this change was 2.09% (95% CI, 0.21% - 4.0%; P = .03); for the end of the scenario-based training period, it was 3.2% (95% CI, 1.18% - 5.22%; P = .003). In the six-month period after the additional training until the study end, the rate of 12-lead acquisition showed a non-significant relative decrease of 0.15% per week (95% CI, −0.3% to 0.003%; P = .06).

### Secondary Outcome: Effects of Period and Gender on Acquisition Rates

**Figure 2.**
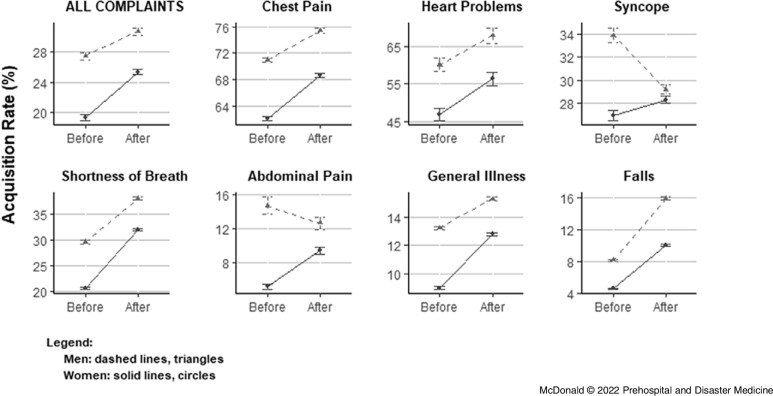
Mean Rates of 12-Lead Acquisition. Note: Percent mean rates of acquisition (all Y axes), before and after interventions, in both all complaints and individual categories. Error bars denote 95% confidence intervals.

Table 2 summarizes the differences in acquisition rates between period and gender. There were significant main effects for both factors: 12-leads were acquired more often in the period after training in most categories (except syncope and abdominal pain), and they were acquired less often in women than men in all. The significant positive interactions illustrated the additional combined effect of period and gender: rate increases in the after period were greater among women than men, both overall (2.8%; 95% CI, 1.9% - 3.6%; P < .0001) and in all complaint categories except falls and heart problems. In the case of falls, the negative interaction term indicated a greater effect among men (−2.3%; 95% CI, −2.5% to −2.0%; P < .0001), whereas a non-significant finding among heart problems showed no additional change beyond the main effects of period and gender (1.6%; 95% CI, −2.0% to 5.3%; P = .38). These results are shown graphically in Figure 2 in terms of mean rates of 12-lead acquisition among men and women in the before and after periods. In most cases, the difference between men and women narrowed, while it widened in the case of falls and stayed the same in heart problems. This figure also illustrates the relative contribution of each factor. In the cases of syncope and abdominal pain, for example, the decreasing rate of acquisition among men contributed to the narrowing difference between men and women.

**Table 2.** Percent Differences in Rates of 12-Lead Acquisition, by Period and Gender

		Main Effects		Interaction
Complaint	Candidates for Assessment (Count)	Period (After minus Before)	Gender (Women minus Men)	Additional Change in Rate due to Combined Effect of Period and Gender^a^ (Women, After minus Men, Before)
All Complaints	37068	3.2 (2.6, 3.9)	−8.1 (−8.7, −7.4)	2.8 (1.9, 3.6)
Chest Pain	5188	4.4 (3.9, 4.9)	−8.8 (−9.3, −8.4)	2 (1.3, 2.7)
Heart Problems	1114	7.8 (5.1, 10.6)	−13 (−15.5, −10.6)	1.6 (−2, 5.3)^b^
Syncope	4358	−4.7 (−5.3, −4.1)	−6.9 (−7.6, −6.3)	6 (5.1, 6.9)
Short of Breath	5582	8.4 (8, 8.8)	−8.9 (−9.3, −8.6)	3 (2.5, 3.5)
Abdominal Pain	1992	−2.0 (−3, −1.1)	−9.5 (−10.3, −8.6)	6.3 (5.1, 7.4)
General Illness	10342	2.1 (1.9, 2.2)	−4.2 (−4.4, −4.1)	1.8 (1.5, 2)
Falls	8492	7.7 (1.5, 7.9)	−3.6 (−3.8, −3.4)	−2.3 (−2.5, −2)

Note: All percent difference values 95% CI.

a Positive interactions indicate increased rates among Women in the After period compared to Men, Before.

b Associated P value = .38. All others < .0001.

## Discussion

Within this urban EMS agency, the introduction of a revised protocol and follow-up training were both associated with modest increases in the rate of 12-lead ECG acquisition among patients with qualifying complaints. Within these findings, differences in rates of ECG acquisition between women and men in most complaint categories were reduced but not eliminated in the time period after the QI interventions. In line with an increasing number of studies that interrogate a wide range of health care settings and practitioners for unconscious bias,^29–31^ the current study was designed to illuminate a previously unexamined area of practice. The results presented here showing a persistent gender gap oblige providers in this service to reflect on their choices and confront their assumptions in these specific situations. They also encourage the same elsewhere. These data amount to a powerful training tool that does more than simply confirm that differences in care exist: it exposes unconscious bias so that it can be consciously addressed.

This study’s findings align with the limited data on gender differences in 12-lead acquisition in prehospital care. One recent retrospective database study of patients with diagnosed myocardial infarctions from Australia found 12% lower odds for women receiving a prehospital 12-lead as compared to men (OR: 0.88; 95% CI, 0.83 − 0.92).^32^ An unpublished master’s thesis from New Zealand also reported significantly lower odds for women (adjusted OR: 0.50; 95% CI, 0.40 - 0.63).^33^ The latter study reported the rates of acquisition (men: 49.6%; women: 34.1%), but patients were included only if presenting with complaints of chest pain and treated with a working diagnosis of ACS by attending paramedics.^33^ While acknowledging differences in methods, geography, and time, the results of the current study (where at least 60% of patients with chest pain received a 12-lead) are comparable when considered on similar terms. Overall rates of acquisition among patients with qualifying complaints (reported here in the range of 20% - 30%), while relatively low, should be interpreted in the context of this study’s aim and design. The QI project sought to increase rates of acquisition using the broadest indications for assessment, acknowledging that measured outcomes will over-count cases where ACS was considered and therefore under-estimate acquisition rates among those assessed. Under these conditions, the results nevertheless show significant improvement. Additionally, the methods of inclusion (particularly using MPDS criteria) establish a consistent and reproducible basis for comparison that could be applied in any service using the same system.

To the authors’ knowledge, no prior studies have analyzed rates of ECG acquisition in terms of presenting complaints other than chest pain. The complaint-based analysis here shows variable rates of acquisition. However, the relevance of these results lies in the changes within each complaint over time rather than the absolute rates compared across categories: it is unsurprising that 12-leads were acquired more often in cases that were dispatched as chest pain calls as compared to general illness. Results for each complaint have therefore been presented individually. These findings signal strongly both that rates improved after training and that the gender gap decreased but did not disappear. There is no obvious explanation for the decreased rates of acquisition among men in calls coded as syncope and abdominal pain. Results in these areas continue to be monitored.

Other relevant literature in this area includes a range of studies that have examined out-of-hospital cardiac arrest/OHCA in terms of gender. Many of these report that women receive standard interventions less often or later as compared to men.^9,26,34–36^ In a North American context, some of these studies have confirmed that women are resuscitated or survive to discharge less frequently,^26,34,36,37^ although these findings have not been seen in other settings in Europe.^38,39^ A QI project in North Carolina (USA) reported decreased differences in many interventions (such as bystander cardiopulmonary resuscitation) between genders, but did not see those effects translated to outcomes.^36^

The results presented here can be placed in the context of the goals of the QI project and the local system. At its outset, the project aimed to increase the percentage of patients with possible ACS who receive a 12-1ead ECG to 50% within 12 months. This goal was not met. In the absence of prior literature and detailed knowledge of ideal rates of acquisition (either overall or in particular complaint categories), the initial target was admittedly arbitrary. Having quantified outcomes in this study, future work could potentially target more relevant and achievable thresholds based on specific complaints. This project also faced additional obstacles common to many QI initiatives in EMS.^40^ These include chronic challenges in balancing training with operational demands, as well as resource limitations in the available training time and personnel.

Although the improvements did not reach the pre-specified goal, the observed increases after the interventions represent approximately 36 additional patients per week who received a 12-lead, with more women being assessed in most categories than previously. Results of this project have been distributed within the service and incorporated into regular training. Rates of acquisition continue to be monitored, and additional qualitative research examining provider attitudes towards gender and 12-lead acquisition is on-going. Finally, in these results, the rates of ECG acquisition in both the lead-in and between-interventions periods showed no appreciable trend, increasing the confidence in the effect of the interventions. At the same time, although the decreasing trend in the period after training did not reach statistical significance, it is unlikely that there would be no decay if observed over a longer time. Comparing these trends suggests the possibility that the effect of changing a written protocol persists for longer than in-person training. While many factors contribute to knowledge decay, it might be possible to maximize practice change by structuring a QI initiative around in-person training followed by supporting changes to guidelines. This hypothesis should be investigated further in future QI efforts.

## Limitations

This study is subject to some limitations. The main objective of the study was to assess ECG acquisition rates before and after a QI initiative aimed at inclusivity in prehospital care. The study design limits any interpretation of actual clinical outcomes associated with increased identification of potential ACS cases and 12-lead interpretation. As discussed previously, the wealth of hospital literature demonstrating the gap in morbidity and mortality outcomes related to under-identified ACS points strongly to a likelihood of benefit. The retrospective nature of this study precludes a causal analysis of outcomes. Additionally, ePCR data may be subject to bias due to the necessarily broad inclusion criteria, as well as possible variations in the practice of documenting key interventions. Every effort has been made to account for this variation where possible; to the extent it is present, it would be expected to under-estimate the true rates of acquisition.

## Conclusion

A QI project aiming to increase the rate of 12-lead acquisition in the prehospital setting resulted in an overall increase in 12-leads acquired. Pre-existing gaps in rates of acquisition between men and women were reduced but did not disappear. On-going research is examining the reasons behind these differences from the perspective of prehospital providers. Future work will continue to monitor acquisition rates, link care to patient outcomes, investigate optimal design of QI projects, and seek to leverage these results in on-going training.
